# Identification and Diagnosis of Foetal Alcohol Spectrum Disorder (FASD) in Children at High Risk of Prenatal Alcohol Exposure: A Service Evaluation

**DOI:** 10.1192/bjo.2025.10480

**Published:** 2025-06-20

**Authors:** Holly Dawson, Suvidhi Khurana, Milla McNally, Amanda Waldman, Rani Samuel

**Affiliations:** 1 South London and Maudsley NHS Foundation Trust, London, United Kingdom; 2 Kings College London, London, United Kingdom.; 3 University of Geneva, Geneva, Switzerland

## Abstract

**Aims:** Prenatal alcohol exposure (PAE) is a leading preventable cause of neurodevelopmental, mental health, and cognitive difficulties. This study evaluates the identification of Foetal Alcohol Spectrum Disorder (FASD) in a high-risk cohort of adopted and Children In Care (CIC) of the local authority, referred to Symbol Team, a specialist Tier 3 service for Children Looked After (CLA) and Adopted young people within Lewisham Child and Adolescent Mental Health Service (CAMHS).

**Methods:** Retrospective service evaluation was conducted on 91 children referred to Symbol Team in June 2023, who were assessed for mental health and neurodevelopmental concerns. Children were categorised based on confirmed Prenatal Alcohol Exposure (PAE) and other reported risks, including maternal alcohol misuse, drug misuse, tobacco use, mental health concerns, reduced antenatal care and a family history of substance misuse. Risk factors were identified through various reports including social service records.

Very High Risk (VHR): Confirmed PAE based on parental reports or hair strand tests.

High Risk (HR): Uncertainty regarding PAE but with 5 or more risk factors.

Moderate Risk (MR): Uncertainty regarding PAE with 3–4 reported risk factors.

**Results:** Of the 91 children referred, 58% (n=53) were categorised as high or moderate risk of PAE. Of these, 16% (n=15) were Very High Risk (VHR), 18% (n=16) were High Risk (HR), and 24% (n=22) were Moderate Risk (MR).

Neurodevelopmental concerns were high across categories, with symptoms related to Autism Spectrum Disorder (ASD) in 43% (n=39) and Attention-Deficit/Hyperactivity Disorder (ADHD) in 46% (n=42). In the VHR group ASD 14% (n=13) and ADHD 15% (n=14) concerns overlapped and similarly the HR group exhibited ASD 11% (n=10) and ADHD 12% (n=11).

Cognitive difficulties were reported by 52% (n=47) of children, with 15% (n=14) in VHR, 15% (n=14) in HR, and 21% (n=19) in MR groups. Mental health concerns like depression were seen in 2% (n=2) VHR, 2% (n=2) HR and 3% (n=3) MR groups. A total of 33% (n=30) received medication treatment, with 11% (n=10) VHR, 8% (n=7) HR and 14% (n=13) in MR groups.

**Conclusion:** Despite the high prevalence of neurodevelopmental concerns and risk factors, FASD identification and diagnosis rates were low. The study also highlights gaps in knowledge, diagnostic tools and clinician training across agencies. Some barriers include under-reporting and stigma associated with the diagnosis. Improved access to reliable records and standardised diagnostic pathways are needed to facilitate early identification of FASD in at-risk children.

